# Optimal velocity loss threshold for inducing post activation potentiation in track and field athletes

**DOI:** 10.5114/biolsport.2023.119284

**Published:** 2022-09-06

**Authors:** Zihang Yuan, Kaifang Liao, Yumei Zhang, Mengyuan Han, Chris Bishop, Zhili Chen, Xiaohua Zhang, Guochao Zhang, Yongming Li

**Affiliations:** 1 School of Physical Education and Sport Training, Shanghai University of Sport, Shanghai, China; 2 Hangzhou No.2 High School of Zhejiang Province, Zhejiang, China; 3 Faculty of Science and Technology, London Sport Institute, Middlesex University, London, United Kingdom; 4 China Institute of Sport Science, Beijing, China

**Keywords:** Counter movement jump, Jump height, Peak power output, Momentum, Squat

## Abstract

The aim of this study was to determine the optimal velocity loss (VL) threshold that maximises the post activation potentiation (PAP) stimulus for achieving larger and more consistent performance gains in track and field athletes. Twenty-two athletes from athletics participated in four back squat PAP tests with four different VL threshold (5%, 10%, 15% and 20% VL) at an intensity of 85%1RM. Countermovement jump (CMJ) height, power, and momentum were assessed before, and 10 s, 4, 8, 12, 16 minutes after the PAP condition. Repetitions of the squat in all the PAP conditions were also recorded. Only the 5% VL condition produced significant improvements in height (ES = 0.73, P = 0.038), peak power output (ES = 0.73, P = 0.038) and momentum (ES = 0.72, P = 0.041) of CMJ, and these changes appeared 8 minutes after the condition. The total number of repetitions during the 5% VL condition was significantly lower than that observed in the 15% (P = 0.003) and 20% VL (P < 0.001) trials. The results from this study indicate that 5%VL during the 2 sets preconditioning squat at 85%1RM was optimal for eliciting PAP in a CMJ exercise, and resulted in significant increases at the 8-min recovery period. The same squat condition also had the least number of repetitions. However, considering the efficiency in practice, athletes can also choose the rest time of 4-min, which can also achieve similar results.

## INTRODUCTION

Post-activation potentiation (PAP) is a physiological phenomenon characterized by the acute increase of muscle force, speed or explosive force after maximal or near maximal intensity resistance exercise [1]. PAP has attracted wide attention as a training and research phenomenon. Previous studies have shown the acute and chronic effects of PAP either in the form of a warm-up [2–4] or contrast training [5–7]. The effects of PAP depend on the mutual relationship between the enhancement and fatigue induced by a pre-loading stimulus [8]. Evidence indicates that only when the enhancement is greater than the fatigue, will PAP be induced, and transiently improve subsequent performance [8].

Traditionally, percentage of maximum repetition (%1RM) and fixed repetitions [9] have been applied to prescribe the load used in any given training program. However, these methods may have some inherent drawbacks owing to individual variability of fatigue at any given number of repetitions or fixed intensity [10]. Moreover, the maximum strength of the individual will fluctuate from day to day, due to the influence of nutrition, exercise and circadian rhythm [11]. As such, the use of %1RM may attenuate any possible enhancements in performance due to a mismatch between the target intensity and the actual intensity needed by the individual at any given time [12]. Consequently, the individual effects of PAP via these traditional methods may lead to a lack of enhancement or excessive fatigue.

Recently, there has been increasing evidence showing that velocity-based training (VBT) is effective in controlling fatigue levels and accurately estimating resistance load in real time [12, 13]. Weakley et al. [14] found that under the same load, with increasing levels of velocity loss (VL), the concentration of RPE and blood lactate at the end of strength training gradually increased, while vertical jump height gradually decreased. Thus, it could be argued that VL is just as an effective method when prescribing training programs, with the intention of avoiding excessive fatigue and achieving the same level of acute stimulation [13], compared with the aforementioned traditional methods. We speculate that different VL thresholds with the same intensity will produce different training effects, with 20–40% VL more conducive to muscle hypertrophy, and < 20%VL more conducive to the development of strength [15–17]. However, with a distinct lack of evidence in this specific area, it remains unclear whether the use of VBT is a viable method for inducing a PAP stimulus in athlete populations.

The traditional PAP condition measures the load by the percentage of maximum repetition (%1RM) and a fixed number of repetitions. This ignores the individual variability of fatigue, which can subsequently impact whether PAP occurs. Therefore, the present study aimed to compare four different VL (5%, 10%, 15% and 20%) conditions to determine the optimal VL threshold which could produce the best PAP response. Given that 5% VL is likely to generate less fatigue than 10–20% VL [14, 18], our hypothesis was that the 5% VL would be the optimal VL threshold in comparison with the other conditions. Our results would help practitioners better prescribe training programs using VBT methods, with the intention of providing an acute PAP response.

## MATERIALS AND METHODS

### Experimental Approach to the Problem

This study adopted a repeated measures randomized cross-over design to evaluate the PAP effects of squatting on CMJ performance, while using four different VL thresholds (5%, 10%, 15% and 20%). All subjects initially participated in a 1RM back squat test and four PAP squat tests with a minimum of 48-h apart, but at the same time of the day. Each PAP test corresponded to one of the four VL thresholds. CMJ performance was assessed prior to and post each PAP condition.

### Subjects

Twenty-two male athletes from athletics (high jump, long jump, three step jump, discus, hammer, 110-meter hurdles, 400-meter hurdles, 100-meter race, 200-meter race, 400-meter race) (Table 1) volunteered to participate in this study in their respective off-season, with written informed consent provided by all. All subjects were clear of cardiovascular or respiratory disease, and free from injury at the time of testing. All subjects were experienced in squat exercises (5.8 ± 2.6 years). Any strenuous activities such as resistance or plyometric training were refrained for at least 48-hours before each testing session. The study was approved by the local ethics committee.

**TABLE 1:** Physical characteristics of subjects at baseline (*n* = 22)

Variables	Mean ± SD
Age (years)	21.1 ± 2.0
Height (cm)	180.3 ± 6.1
Body mass (kg)	75.1 ± 9.9
Resistance training experience (years)	5.8 ± 2.6
1RM (kg)	144.3 ± 22.9
1RM/ body mass	1.9 ± 0.2
Body fat percentage (%)	13.8 ± 4.3

RM = repetition maximum

### Procedures

#### 1RM squat test

On the first visit, the subjects had their body mass, height and body fat percentage measured, and performed the 1RM free-weight barbell back squat test. After a standardized warm-up (a five-minute run, dynamic stretching for lower extremities, two 20-meter sprints and three CMJs) the subjects started the test with 40 kg. The test weight was progressively increased with an increment of 20 kg until that the attained mean velocity (MV) was lower than 0.5 m/s. Back squat took the standard technique as described in the NSCA textbook [19], whereby subjects held the barbell in a closed pronated grip, with the barbell positioned on the upper trapezius muscle. Initial posture encouraged participants to position their feet approximately shoulder width apart, with slight external rotation of the feet. Participants were instructed to descend to a depth where the upper thighs were parallel to the ground and then extend the hips, knees and ankles to the initial posture as quickly as possible. Thereafter, the weight was increased with smaller increments (i.e. 5 to 1 kg) individually for each subject, so that 1RM could be determined with precision. The weight that each subject could squat only once with standard technique was considered to be their 1 RM. Trained spotters were present on both sides of the barbell during the squat to ensure safety. Three, two, and one attempts were executed at light (i.e. MV > 0.7 m/s), medium (0.7 m/s ≤ MV ≤ 0.5 m/s), and heavy (MV < 0.5 m/s) loads, respectively. Rest periods were 4-minutes for the light and medium loads, and 6-minutes for the heavy load.

#### PAP test

**FIG. 1:**
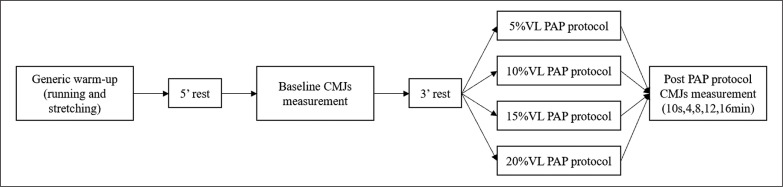
Schematic representation of PAP test PAP, post activation potentiation; CMJ, counter movement jump.

The following four visits were for the four PAP conditions which were conducted in a counterbalanced randomized order (Figure 1). During each visit, subjects started with a standardized warm-up, which was the same as the 1RM test. After 5-minutes of rest, subjects performed three CMJs and chose the best as the baseline level. Afterwards, the subjects rested for 3-minutes before performing two sets of squats at 85%1RM [20] with 1-minute rest [21, 22] in between as the PAP condition. In the PAP condition, a 5-cm wide line was marked parallel to the barbell bar and set at 30-cm behind the barbell lever. Subjects were required to place their toes on the line, with their foot as wide as the shoulder (or wider), with toes slightly faced outward. One elastic band with 1-m length was set directly 50–70 cm distance away from the mark line. The height of the band was equal to the squatting depth with thigh parallel to the ground. A GymAware Powertool tachometer (Kinetic Performance, Canberra, Australia) was placed on the floor at right side of the squatting rack with the cable aligned perpendicular to the ground to monitor the velocity of each repetition in real-time. All subjects were verbally encouraged to perform the concentric phase of the lift as ballistically as possible, with real-time auditory feedback of the mean velocity for each repetition. The VL thresholds were calculated according to the following formula: [stop velocity = initial speed × (1-VL)] [10]. Sets were terminated when the repetition velocity was recorded below the target velocity of each specific zone. The PAP condition design is based on the finding that multiple sets and the high-intensity (≥ 85%1RM) will get better effect of PAP [20], and on the basis of past research to determine between set rest periods of 1-minute [6, 32]. After the two sets of squats, subjects conducted CMJ testing at 10-s, 4, 8, 12, and 16-minutes of the recovery period.

#### CMJ test

Each assessment of CMJ included 3 trials with 10 s rest between each trial. During each trial, the subjects were in an upright initial position with their hands on hips and their feet approximately shoulder width apart. After hearing the instruction to “jump”, athletes performed a countermovement to a self-selected depth (to avoid alterations in jump strategy), before performing an explosive vertical jump. During the flight phase, subjects were required to keep their legs straight and hands on hips throughout [16]. The best of the three trials was chosen for subsequent analysis. Performance variables assessed during the CMJs included jump height, peak power output (PPO) and jump momentum (calculated by multiplying take-off velocity by body mass) [23] using a jump mat (Smartjump, Fusion Sport, Australia). Verbal encouragement was given throughout all CMJs assessments. Room temperature was maintained between 20 and 24°C.

#### Statistical Analyses

Data were expressed as the mean ± SD. Jump heights, PPOs and momentum in CMJs were then analyzed using a 4 × 6 (VL × assessment time) factorial analysis of variance (ANOVA) with repeated measures. The alpha level was set at p < 0.05, and the Huynh-Feldt adjustment was used where required based on a test of sphericity. One-way ANOVA was used to analyze the repetitions of PAP condition. If the homogeneity of variance was satisfied, Tukey test was used for pairwise comparison. Games-Howell test was used to compare the results. Paired samples t-test and Cohen’ d effect size were also used to detect the standardized mean differences of jump height, PPO, momentum between the baseline and other time points and the total number of CMJ repetitions between different VL sets. The criteria to interpret the strength of the effect size was as follow: trivial (< 0.2), small (0.2~0.6), moderate (0.6~1.2), large (> 1.2) [24].

## RESULTS

### Jump Height, Peak Power Output and Jump Momentum

A significant time effect of jump height [F_(7.854,164.930)_ = 2.303, P = 0.024], PPO [F_(7.997,167.946)_ = 2.415, P = 0.017] and jump momentum [F_(1.868,39.224)_ = 11.024, P < 0.001] were found between pretest and posttest in all four PAP conditions (i.e. 4 VL) (Table 2). Post hoc analyses found that at the 10-s of recovery, compared with the baseline, the jump height, PPO and momentum did not change significantly in the 5% VL condition (P > 0.05), but decreased significantly in the 10% VL (P < 0.001), 15% VL (P < 0.001) and 20% VL (P < 0.001) condition. At the 8-minute time point, only 5% VL had significant increases compared with baseline data for jump height (ES = 0.73, P = 0.038), PPO (ES = 0.73, P = 0.038) and momentum (ES = 0.72, P = 0.041), while 10% VL (P > 0.05), 15% VL (P > 0.05) and 20% VL (P > 0.05) had no significant changes. There was no significant difference between the jump height, PPO and jump momentum of each VL and the baseline at the 4, 12 and 16-minutes of the recovery (P > 0.05) (Table 3).

**TABLE 2:** Jump height, PPO and momentum of CMJ during PAP condition of different VL (n = 22).

CMJ performance variables	VL (%)	Assessment time point					
		Baseline	10 s	4 min	8 min	12 min	16 min
Height (cm)	5	53.43 ± 6.52	51.89 ± 5.95	55.03 ± 7.40	54.88 ± 7.12*	53.98 ± 7.45	53.79 ± 7.09
	10	54.57 ± 6.44	51.13 ± 5.51*	55.12 ± 6.74	55.11 ± 6.65	54.66 ± 6.22	54.08 ± 6.96
	15	54.49 ± 5.66	50.92 ± 6.00*	55.75 ± 6.70	55.49 ± 6.81	54.59 ± 6.93	53.81 ± 6.41
	20	54.43 ± 6.78	50.26 ± 6.12*	55.77 ± 6.45	55.32 ± 6.52	54.74 ± 7.02	54.31 ± 6.25

PPO (W)	5	4589.95 ± 550.54	4496.62 ± 530.30	4686.86 ± 565.86	4677.89 ± 558.92*	4623.34 ± 577.29	4611.69 ± 554.19
	10	4659.42 ± 568.89	4450.48 ± 545.85*	4692.58 ± 569.90	4691.74 ± 547.83	4664.66 ± 540.95	4624.32 ± 582.49
	15	4640.77 ± 479.06	4422.48 ± 495.19*	4713.67 ± 521.57	4714.35 ± 534.90	4658.20 ± 534.63	4604.77 ± 512.70
	20	4650.44 ± 491.68	4397.39 ± 492.40*	4732.04 ± 488.99	4704.58 ± 525.83	4669.83 ± 529.96	4643.29 ± 511.43

Momentum (N · m)	5	242.34 ± 32.30	238.90 ± 31.22	245.71 ± 31.93	245.45 ± 32.29*	243.37 ± 32.49	242.95 ± 31.80
	10	245.08 ± 33.90	237.37 ± 32.88*	246.22 ± 33.60	246.09 ± 32.55	245.20 ± 32.86	243.50 ± 33.83
	15	244.18 ± 29.94	235.81 ± 29.02*	246.79 ± 31.00	246.78 ± 31.56	244.66 ± 31.32	242.74 ± 30.37
	20	244.14 ± 29.09	234.70 ± 28.82*	239.42 ± 45.34	246.44 ± 31.24	245.19 ± 30.73	244.34 ± 30.72

* —significantly different from baseline. PPO, peak power output; PAP, post activation potentiation; CMJ, counter movement jump

**TABLE 3:** The mean differences between baseline and other time point in Jump height, PPO and momentum

CMJ performance variables	VL (%)	Effect size with 95%CI				
		10 s	4 min	8 min	12 min	16 min
Height (cm)	5	0.56 (0.10, 1.01)	0.69 (0.21, 1.15)	0.73 (0.25, 1.20)	0.23 (-0.20, 0.65)	0.15 (-0.27, 0.57)
	10	1.67 (1.01, 2.32)	0.33 (-0.10, 0.76)	0.26 (-0.17, 0.68)	0.04 (-0.38, 0.46)	0.15 (-0.27, 0.57)
	15	1.29 (0.71, 1.86)	0.59 (0.13, 1.04)	0.41 (-0.03, 0.84)	0.04 (-0.38, 0.46)	0.27 (-0.16, 0.69)
	20	1.53 (0.90, 2.15)	0.69 (0.22, 1.15)	0.42 (-0.03, 0.85)	0.12 (-0.30, 0.54)	0.04 (-0.38, 0.46)

PPO (w)	5	0.56 (0.10, 1.01)	0.69 (0.21, 1.15)	0.73 (0.25, 1.20)	0.23 (-0.20, 0.65)	0.15 (-0.27, 0.57)
	10	1.67 (1.01, 2.32)	0.33 (-0.10, 0.76)	0.26 (-0.17, 0.68)	0.04 (-0.38, 0.46)	0.18 (-0.25, 0.60)
	15	1.29 (0.71, 1.86)	0.58 (0.12, 1.03)	0.41 (-0.03, 0.84)	0.04 (-0.38, 0.46)	0.27 (-0.16, 0.69)
	20	1.53 (0.90, 2.15)	0.69 (0.22, 1.15)	0.42 (-0.03, 0.85)	0.12 (-0.30, 0.54)	0.04 (-0.38, 0.46)

Momentum (N · m)	5	0.53 (0.08, 0.97)	0.64 (0.18, 1.10)	0.72 (0.25, 1.19)	0.19 (-0.23, 0.61)	0.11 (-0.31, 0.53)
	10	1.85 (1.14, 2.53)	0.31 (-0.12, 0.74)	0.22 (-0.21, 0.64)	0.02 (-0.40, 0.44)	
	15	1.31 (0.72, 1.87)	0.59 (0.13, 1.04)	0.48 (0.03, 0.92)	0.08 (-0.34, 0.50)	0.26 (-0.17, 0.68)
	20	1.65 (1.00, 2.29)	0.13 (-0.29, 0.55)	0.45 (0.00, 0.88)	0.18 (-0.24, 0.60)	0.03 (-0.39, 0.45)

PPO, peak power output; PAP, post activation potentiation; CMJ, counter movement jump

### Number of squat repetitions

The number of squat repetitions in the 2 sets of squats to the 5% VL was significantly less than those to 15% VL (P = 0.003), and 20% VL (P < 0.001), and 20% VL was significantly more than 10% VL (P < 0.001) and 15% VL (P = 0.002) (Table 4).

**TABLE 4:** The total number of repetitions in the first and second group of 4 VL conditions

VL(%)	Number of Repetitions		
	Set 1	Set 2	2 sets in total
5	3.2 ± 1.3^a^	2.3 ± 1.6^a^	5.6 ± 1.9^a^
10	4.1 ± 1.3^a^	3.6 ± 1.5^a1^	7.6 ± 2.2^a1^
15	4.7 ± 1.7^a^	4.2 ± 2.6^b^	8.9 ± 2.9^b^
20	6.8 ± 2.8^b^	5.6 ± 3.0^b1^	12.4 ± 4.6^c^

Note: a, b and c are significance symbols, and the values with these symbols are significantly larger or smaller according to the relationship of a < b < c, p < 0.05. There is no significant difference between a1 and a, b, but it is significantly less than b1.

In set 1, squats to the 5% VL were significantly less than those to 20%VL (P < 0.001), and 20% VL were significantly more than 10%VL (P < 0.001), 15%VL (P = 0.002). In set 2, squats to the 5% VL were significantly less than those to 15% VL (P = 0.040) and those to 20% VL (P < 0.001), and 20% VL were significantly more than 10% VL (P = 0.023).

## DISCUSSION

The aim of this study was to determine the optimal VL threshold (i.e. 5%, 10%, 15% or 20% VL) in the back squat at 85%1RM to induce the best PAP effect. The primary findings were that 5% VL threshold resulted in significant increases in CMJ height, PPO and momentum at the 8 min recovery period. The same squat condition also had the least number of repetitions.

This study was among the first to explore the optimal velocity loss threshold for inducing PAP. The findings showed that jump height, momentum and PPO of the subjects increased significantly only after the PAP condition with 5% VL. It is found that muscle contraction produces both enhancement and fatigue, and the PAP effect can be induced only when the enhancement exceeds fatigue [1]. Hamada et al. [25] believed that excessive activation would lead to excessive fatigue effect, and then inhibited the emergence of any PAP effect. Therefore, taking appropriate load to avoid excessive fatigue effect is of great significance to the appearance of PAP effect. Gonzalez-Badillo et al. [10] found that there was a strong correlation(r^2^ = 0.83, SEE = 0.09)between VL and the percentage of repetitions (the number of repetitions completed / the maximum number of repetitions). Weakley et al. [14] found that under the same load, the RPE and concentration of blood lactic acid increased with the increase of VL, while the immediate jump height was the opposite, which indicated that the fatigue effect increases with the increase of VL. In the present study, 10 s after the end of 10%, 15% and 20% VL conditions, the jump height, momentum and PPO of CMJ decreased significantly, the fatigue effect is significant, excessive fatigue finally suppresses the appearance of PAP effect. However, after 10 s of 5% VL condition, the jump height, momentum and PPO were not significantly lower than that of the baseline, and there was no significant fatigue in the subjects, the PAP effect was successfully induced. According to the statistics of the squatting repetitions of the subjects in the experiment, it was found that the difference in load caused by different VL conditions may affect the PAP effect. The total repetitions of the 15% VL and 20% VL conditions were significantly higher than that of the 5% VL condition. Additional load (leading to heightened VL) may contribute to fatiguing effects, which may ultimately suppress the appearance of the PAP effect – something that 5% VL condition did not do. In addition, compared with 10% VL, 15% VL and 20% VL conditions, 5% VL condition resulted in fewer squatting repetitions, so it may also have advantages relating to time-efficiency during training. In summary, it is recommended that practitioners use 5% VL condition to induce the PAP effect. It was found that there were individual differences among the subjects, which was also reflected in the number of squatting repetitions. Furthermore, traditional PAP methods may not be sensitive enough to expose such individual differences. However, using VL as a method to monitor training within sessions is likely to be able to better take into account such individual differences, whilst also achieving the same level of acute stimulation [13]. Meanwhile, it has been proved that the relationship between VL and the percentage of completion repetitions is not affected by exercise level [26] and strength growth [27]. However, these two factors can impact the PAP effect [28]. Therefore, the conclusion of this experiment may be applicable for other athlete populations.

In this study, PAP effect was induced at the 8-minutes of the recovery period after the 5% VL condition, which was similar to that of previous studies [20, 29, 30]. Immediately after PAP condition, the fatigue effect was significantly higher than the enhancement effect, resulting in a decrease in exercise performance, but with the extension of rest time, the muscle enhancement was gradually higher than the fatigue, the PAP effect began to appear and gradually reached the optimal interval time, and muscle contraction marks began to have a positive effect on the subsequent explosive force performance until the PAP effect disappeared [31]. There have been several meta-analyses [20, 29, 30, 32] to explore the optimal interval time of PAP effect. Gouvêa et al. [29] reported that rest intervals of 8 to 12 minutes produced the greatest effect (ES = 0.24). Wilson et al. [30] reported rest intervals of 7 to 10 minutes as the most optimal (ES = 0.70). The results of meta-analysis will be affected by inclusion criteria and the division of intermittent periods, but combined with the results of several meta-analyses, the optimal interval time for PAP seems to be 7–12 minutes. However, PAP effect is a highly personalized phenomenon, the optimal interval time may be influenced by individual factors (e.g. fast muscle fiber ratio, strength level) [28]. Therefore, in practical application, coaches need to conduct further research on individuals if they want to know the optimal interval time for athletes accurately, but considering the convenience and universality of practical use, the best PAP effect can be obtained by combining the 5%VL condition with the interval time of 8 minutes. It is worth noting that under the condition of 5% VL, although the height (55.03 ± 7.40, ES = 0.69), PPO (4686.86 ± 565.86, ES = 0.69) and jump momentum (245.71 ± 31.93, ES = 0.64) of 4-minutes were not significantly higher than baseline, they were similar to height (54.88 ± 7.12), PPO (4677.89 ± 558.92) and jump momentum (245.45 ± 32.29) recorded at 8-minutes, and still with a moderate improvement. Considering that practitioners may not always have the time to wait 8-minutes to optimize jump performance in practice, the condition of 5% VL at a 4-minute interval may be a viable alternative, with negligible differences in results.

The study also had some limitations which should be noted. First, this study only explored the optimal VL under 85%1RM, but did not carry out with more load intensity. However, 85%1RM is a common intensity used in resistance training [21, 33–35] and helps to distinguish between different VL conditions. Second, this study investigated only male subjects, but previous studies showed that there was no gender difference in PAP effect [36], so the findings of this study are still applicable to females.

## CONCLUSIONS

The current findings indicate that if practitioners are using VBT to induce PAP effect, it is advised to use 5% VL, and to leave 8-minutes of recovery between the pre-load stimulus and the explosive activity in order to obtain the optimal benefit. If practitioners do not have the time to wait 8-minutes, they can choose the condition of 5% VL at a 4-minutes interval.
